# Identification of germline population variants misclassified as cancer-associated somatic variants

**DOI:** 10.3389/fmed.2024.1361317

**Published:** 2024-03-20

**Authors:** Rebecca D. Pollard, Matthew D. Wilkerson, Padma Sheila Rajagopal

**Affiliations:** ^1^Maret School, Washington, DC, United States; ^2^Metis Foundation, San Antonio, TX, United States; ^3^Center for Military Precision Health, Uniformed Services University, Bethesda, MD, United States; ^4^Department of Anatomy, Physiology and Genetics, Uniformed Services University of the Health Sciences, Bethesda, MD, United States; ^5^Cancer Data Science Laboratory, Center for Cancer Research, National Cancer Institute, Bethesda, MD, United States; ^6^Women’s Malignancies Branch, Center for Cancer Research, National Cancer Institute, Bethesda, MD, United States

**Keywords:** germline, somatic, variant classification, misclassification, health disparities

## Abstract

**Introduction:**

Databases used for clinical interpretation in oncology rely on genetic data derived primarily from patients of European ancestry, leading to biases in cancer genetics research and clinical practice. One practical issue that arises in this context is the potential misclassification of multi-ancestral population variants as tumor-associated because they are not represented in reference genomes against which tumor sequencing data is aligned.

**Methods:**

To systematically find misclassified variants, we compared somatic variants in census genes from the Catalogue of Somatic Mutations in Cancer (COSMIC) V99 with multi-ancestral population variants from the Genome Aggregation Databases’ Linkage Disequilibrium (GnomAD). By comparing genomic coordinates, reference, and alternate alleles, we could identify misclassified variants in genes associated with cancer.

**Results:**

We found 192 of 208 genes in COSMIC’s cancer-associated census genes (92.31%) to be associated with variant misclassifications. Among the 1,906,732 variants in COSMIC, 6,957 variants (0.36%) aligned with normal population variants in GnomAD, concerning for misclassification. The African / African American ancestral population included the greatest number of misclassified variants and also had the greatest number of unique misclassified variants.

**Conclusion:**

The direct, systematic comparison of variants from COSMIC for co-occurrence in GnomAD supports a more accurate interpretation of tumor sequencing data and reduces bias related to genomic ancestry.

## 1 Introduction

With the rapid advances of targeted therapies and associated biomarkers, genetic data is increasingly necessary to facilitate clinical management of cancer (1, 2). Collaborative databases are used by clinicians to help classify variants from molecular testing as associated with malignancy, inherited rare cancer syndromes, or normal population variation (3, 4). As with many genetics efforts, underrepresentation of non-European ancestral populations in these clinical databases is a critical bottleneck to their universal applicability.

Numerous clinical challenges currently arise from the overwhelming overrepresentation of patients of European ancestry in cancer genetics data. These include inadequate training of clinical tools (as observed in the first generation of commercially available polygenic risk scores) (5, 6); less accurate prediction of treatment response for specific populations in clinic (7–9); inadvertent biases against offering available interventions or studies to patients (10); and insufficient representation in precision oncology registries to inform future translational research work (11, 12).

In the context of clinical variant interpretation databases, one such potential issue is the misclassification of variants as somatic (associated with the cancer) when they are, in fact, germline (associated with patients’ ancestral populations). This may occur depending on the reference genome used and can be clinically problematic if misclassified variants are directly relevant to diagnosis, treatment, or prognosis (13). In other words, such variants may be used as an indication for potential treatment when they may not be cancer-specific, or may be accidentally used by oncologists to provide inaccurate prognostic information or molecular pathologists in the course of diagnosis. Misclassified variants are also critical to be aware of in the context of cancer research. Human variant origin (whether germline or somatic) is often a necessary specification in translational oncology studies ranging from drug mechanism of action to inclusion criteria for clinical trials (14, 15).

While some variant callers have advanced filtering of germline variants from tumor-only data using multiple population databases, they require a baseline knowledge of bioinformatics and typically remove germline variants without characterizing more information about the potential germline variants (16). Other efforts have interrogated somatic variants that have been included in population databases (17).

To evaluate this concern, we compared ostensibly somatic variants from the Catalogue Of Somatic Mutations In Cancer (COSMIC) database (18), used to categorize cancer-specific variants, to population-specific variants from the Genome Aggregation Database (GnomAD) (19). We observed that over 92% of the 208 cancer-associated genes in COSMIC had at least one misclassified variant, and that 6,957 variants (0.36% of all variants in COSMIC) were concerning for misclassification. Of these, we found that the African / African American genetic ancestry population in GnomAD contained the most variants associated with misclassification in COSMIC and the greatest number of unique misclassified variants. Our findings emphasize the need for accurate variant classification across populations for clinicians and translational researchers.

## 2 Methods

### 2.1 Reference databases

The Catalogue Of Somatic Mutations In Cancer (COSMIC) contains variants observed in cancers. These variants are aggregated through expert curation via publication review (focused on specific genes / diseases) and tumor genome-wide screening data (18). Variants in COSMIC were obtained using the unified file from v99.

The Genome Aggregation Database (GnomAD) contains variants and allele frequencies collected from over 76,000 individuals. Variants from GnomAD used in this project were obtained from the annotated Linkage Disequilibrium datasets in v2, in which variants were assigned by GnomAD to 8 ancestral populations: African/African American, Latino/Admixed American, Ashkenazi Jewish, East Asian, Finnish European, Estonian, North-Western European and Southern European.

The COSMIC Cancer Gene Census is an ongoing effort by COSMIC to categorize genes that drive cancers (20). The census is updated on an ongoing basis and available with explanations for each gene and its relationship to cancer here: https://cancer.sanger.ac.uk/census.

### 2.2 Data preparation

Data processing and visualization were performed in Python v3.10.7 by leveraging the Pandas library and matplotlib v3.5.3.

The COSMIC dataset was converted from a tab-separated value (.TSV) format to a comma-separated value (.CSV) format using the Pandas library. Reference and alternate allele columns were added by parsing the “CDS” column in Pandas. The. CSV file was partitioned by transcript accessions to generate 34,317 separate files.

For the GnomAD datasets, we generated. CSV files of variants in each ancestral population. We lifted over the coordinates from GRCh37 to GRCh38 and added a “genomic coordinate” column based on the GRCh38 chromosome and position columns.

### 2.3 Data analysis and variant misclassification identification

We iteratively compared each partitioned COSMIC. CSV file and each GnomAD. CSV file based on 3 parameters: genomic coordinate, reference allele, and alternate allele, with misclassified variants defined as matches across both files. These matches were subsequently merged, and duplicate rows deleted, within ancestral populations. Our pipeline systematically quantified the total number of variants and unique genes per ancestral population.

To facilitate a streamlined comparison of all misclassified variants, we merged all 8 ancestral populations into a list of unique variants listed by genomic coordinates, reference (ref) and alternate (alt) allele columns, and allele frequencies per population.

### 2.4 Statistical analysis

Chi-squared testing was used to identify significant differences by population among misclassified variants and genes.

## 3 Results

### 3.1 Cancer-associated genes at greatest risk for variant misclassification

**Figure 1:**
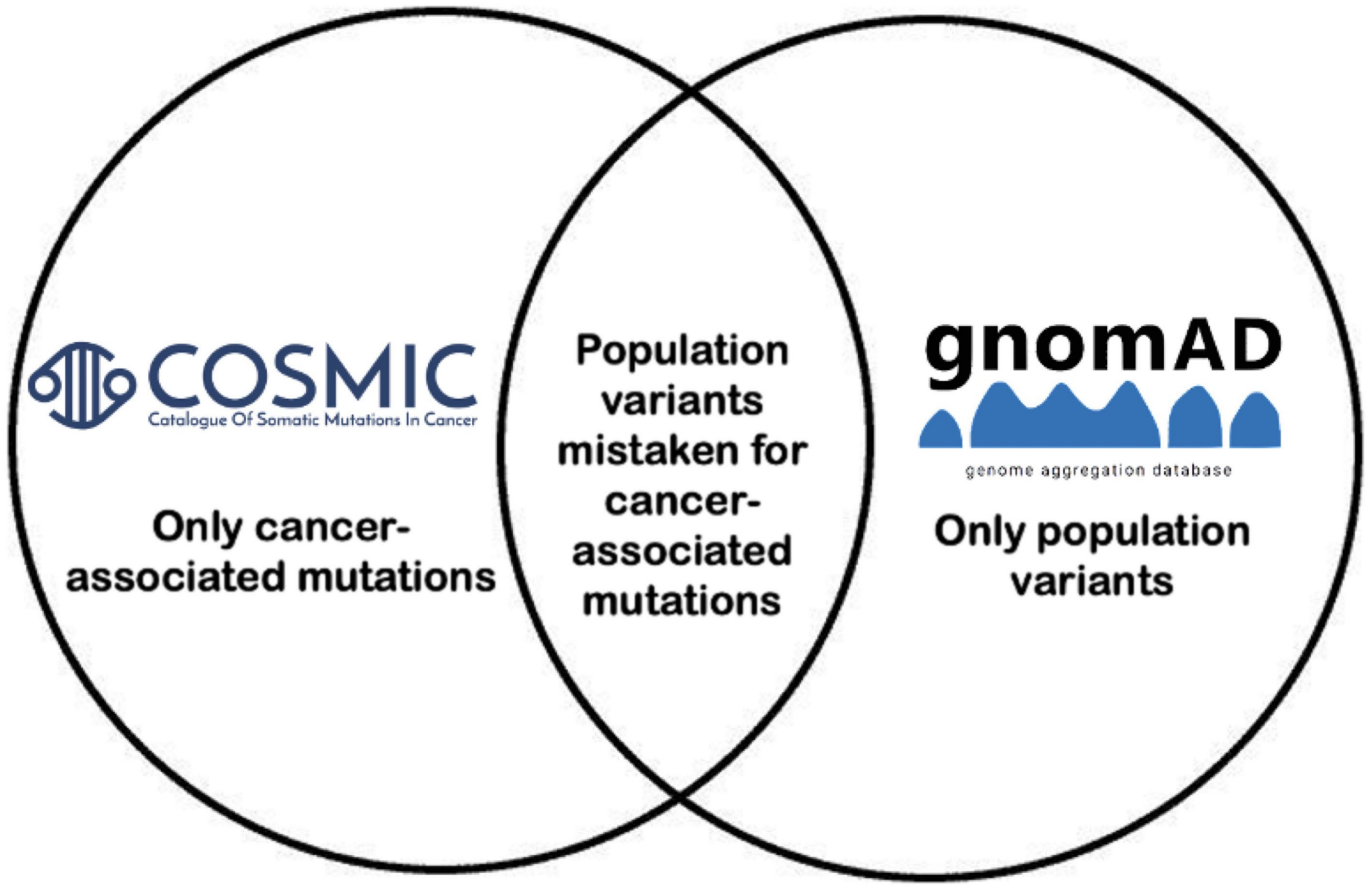
Project overview. Variants that overlapped between COSMIC and GnomAD based on genomic coordinate, reference allele, and alternate allele were incorporated, and variant allele frequencies per population per GnomAD were reported.

Figure 1 demonstrates the project concept. Among 208 cancer-associated genes in the COSMIC cancer gene census, 192 (92.3%) were found to have misclassified variants. *ABL* had the greatest number of unique misclassified variants identified at 274, but 19 genes (*ABL1, PTPRT, HLA-A, JAK2, AFF3, PREX2, EGFR, ETV6, MLLT3, ALK, EBF1, MTOR, NOTCH1, AFDN, KMT2C, FA1, FAM135B, FAT3,* and *MUC4*) were associated with over 100 unique misclassified variants. Supplementary Table S1 lists all 192 genes by number of unique positions in the gene and variants observed.

### 3.2 Frequency of misclassified variants from COSMIC in each ancestral population

**Figure 2:**
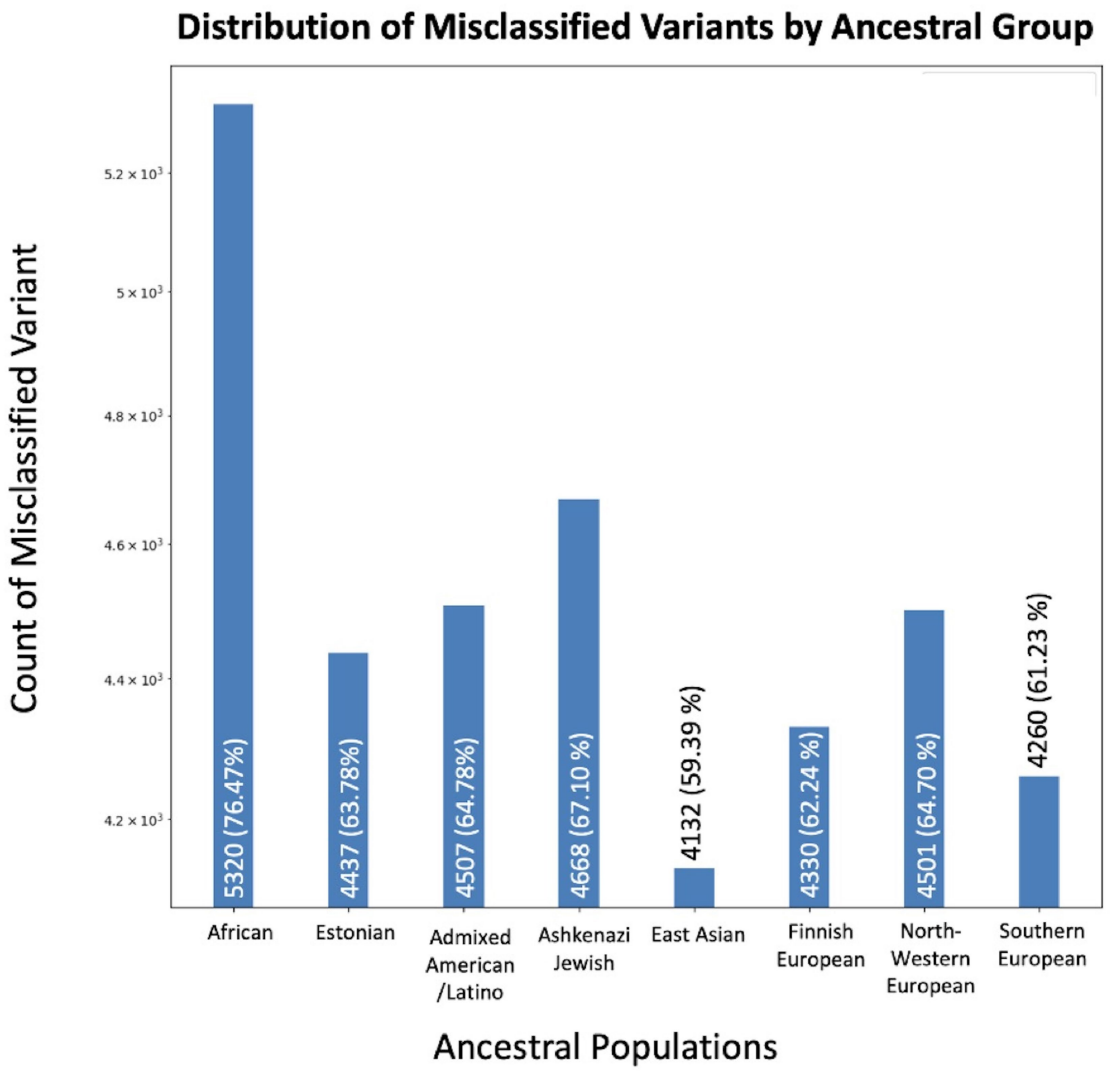
Variants concerning for misclassification in COSMIC by ancestral population. The X-axis displays the number of overlapping variants between COSMIC and GnomAD, while the Y-axis specifies the total count. Variants may be in multiple ancestral groups.

We identified 6,957 unique variants out of 1,906,732 (0.36%) total in COSMIC that aligned with normal population variants in GnomAD (Figure 2). We evaluated how many of these variants were reported in each ancestral population. The population with the greatest inclusion of misclassified variants was the African/African American population, with 5,320 misclassified variants (76.47%). The Ashkenazi Jewish population had the second greatest inclusion of misclassified variants, with 4,668 (67.10%). Comparatively, the other populations included between 59 and 64% of the misclassified variants. The difference of included misclassified variants across populations was statistically significant (*p* < 1×10^−5^).

### 3.3 Proportion of misclassified variants from COSMIC across ancestral populations

**Figure 3:**
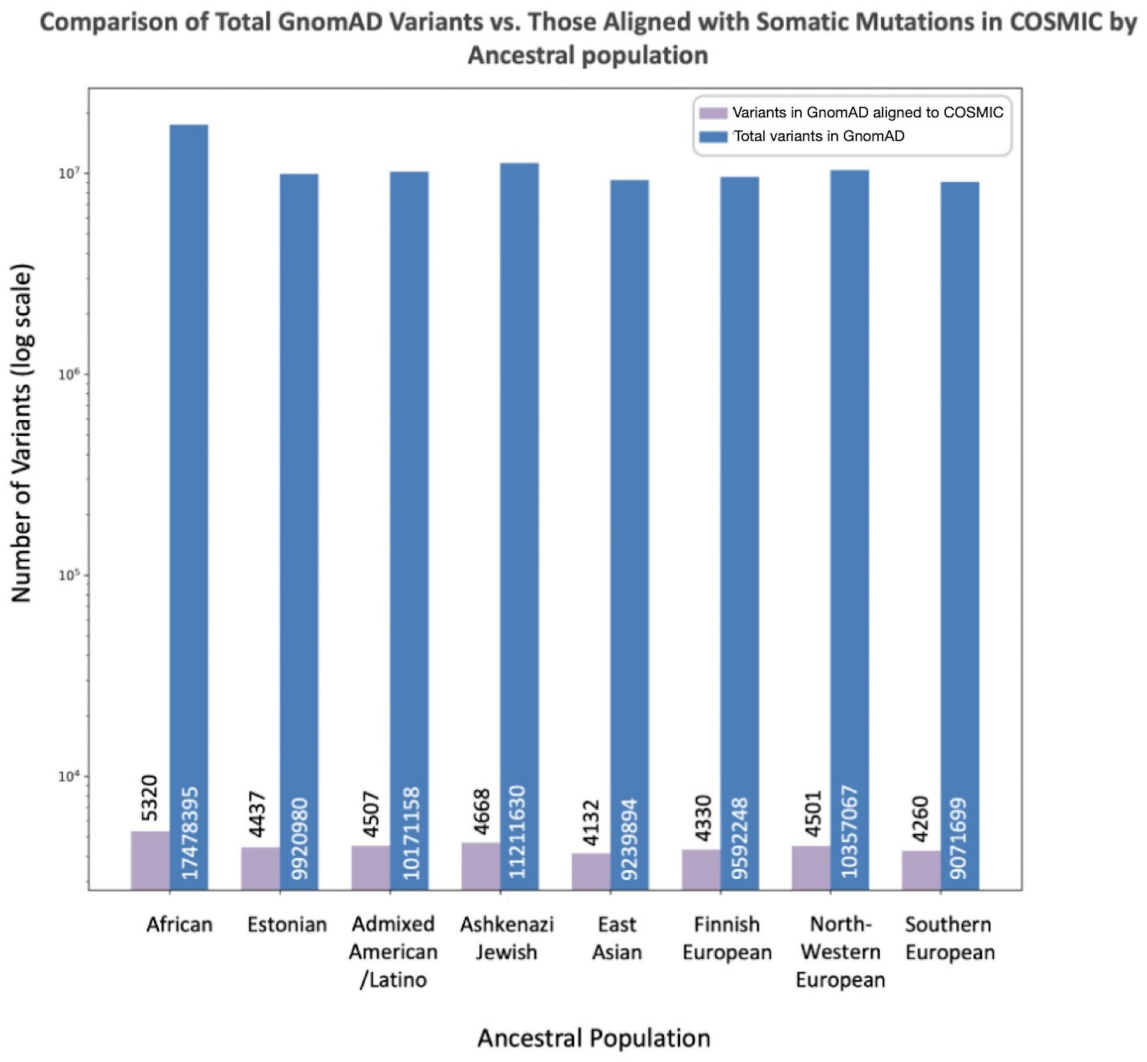
Misclassified variants (aligned between GnomAD and COSMIC) compared to total variants in GnomAD by population. The X-axis lists the specific population, while the Y-axis reports the number of total variants in GnomAD (blue) and number of overlapping variants between COSMIC and GnomAD concerning for misclassification (purple).

To assess the extent to which the total number of variants in GnomAD may influence the number of misclassified variants reported in each population, we compared the number of misclassified variants per population to the overall numbers of variants per population in GnomAD (Figure 3). The African/African American population had the largest number reported variants at 17,478,395, with variants misclassified in COSMIC representing 0.03%. The Southern European population had the smallest number of reported variants at 9,071,699, with variants misclassified in COSMIC representing 0.05%. However, the proportion of misclassified variants across all GnomAD variants per population was not statistically significant.

### 3.4 Unique misclassified variants specific to each population

**Figure 4:**
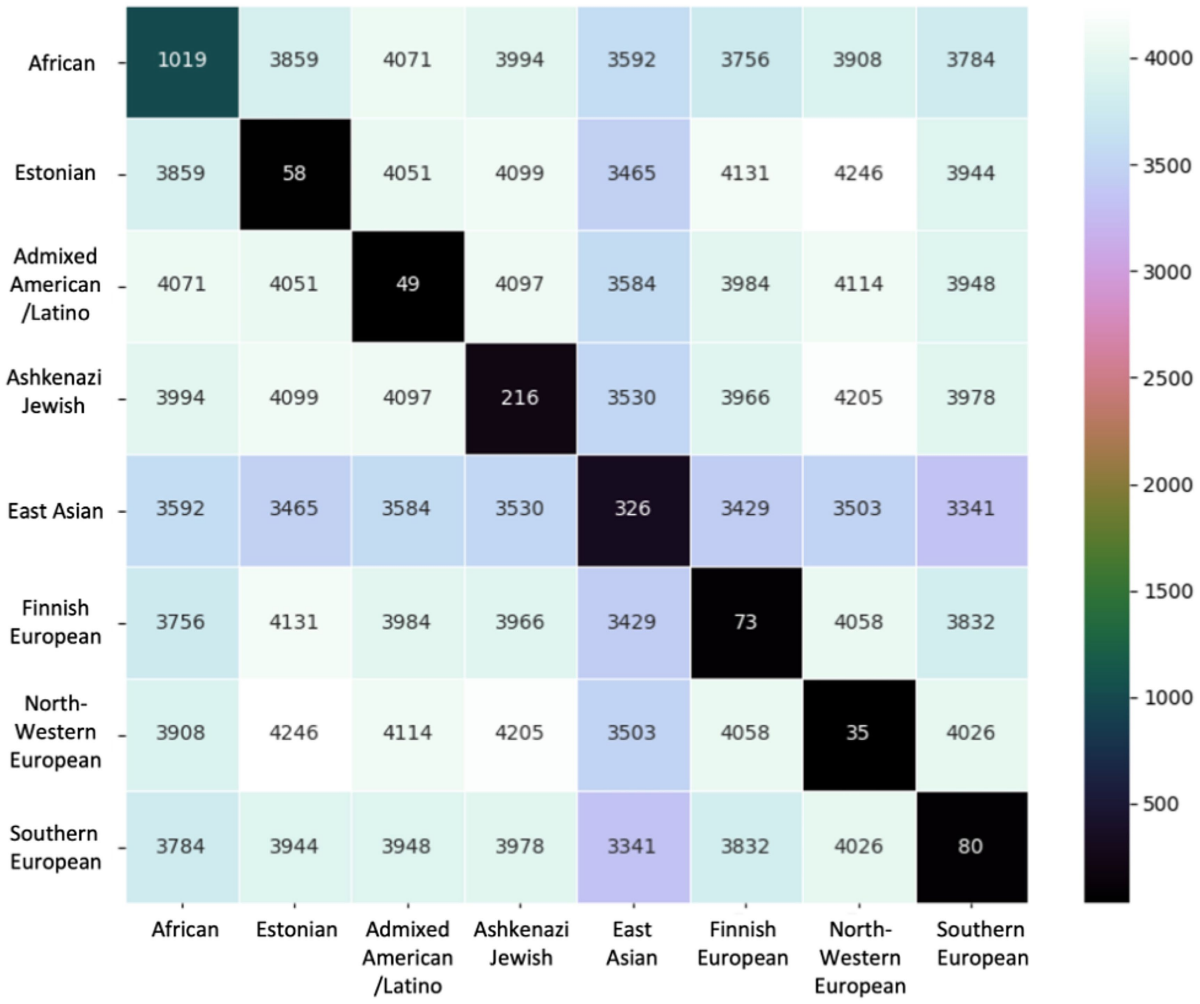
Variants compared pairwise across ancestral groups. The base number of variants is the 6,957 total unique variants identified. Diagonal entries show the number of variants unique to that ancestral group.

We compared shared variants pairwise by population to determine the extent of population-specific versus shared variants across populations (Figure 4). In each pairwise comparison, we reported number shared variants across those populations. We also report the number of variants unique to that population. The African/African American population had more misclassified variants unique to its population (1,019) relative to any other population, followed by the East Asian (326) and Ashkenazi Jewish (216) populations. In contrast, European populations and the Admixed American/Latino population had <100 unique variants.

## 4 Discussion

In this project, we compared variants in the COSMIC cancer gene census to variants in GnomAD across ancestral population to identify potentially misclassified population-level variants. We sought to demonstrate in a straightforward fashion the clinical relevance of our findings by directly comparing across these databases and providing a systematic assessment that reflects the breadth of this issue across an established list of cancer-specific genes.

Although we observed a comparatively low fraction of all variants in COSMIC were affected by potential misclassification, concerningly, we found that over 90 percent of genes in the COSMIC cancer gene census had at least one misclassified variant. Given that COSMIC is routinely used in molecular pathology laboratories to make recommendations for diagnosis, prediction, and prognostication based on cancer-specific variants, it is crucial to identify and address issues that systematically affect accurate variant classification (21).

Among the 19 genes associated with over 100 unique misclassified variants, several are concerning because of variants’ role in prediction of therapy use for patients. *ABL1* mutations, although usually specific to known resistance mutations, can be used to select alternative therapies in chronic myeloid leukemia (18). While *JAK2* V617F and exon 12 mutations are well known to contribute to development of leukemia, other variants are still considered if identified (22). *EGFR* and *ALK* mutations are critically relevant for prediction of treatment response in lung cancer (23). Numerous clinical trials actively seek patients with somatic variants in these genes as well as genes underlying therapeutic targets, such as *mTOR*, to study therapy response. Misclassification is critically important to correctly identifying somatic origin, and accordingly appropriate prediction of patient treatment response, in this setting.

In their documentation, COSMIC reports that they use the Cancer Mutation Census (CMC) as a tool to help users annotate somatic mutations. This effort actually already includes data from ClinVar (germline pathogenic variants associated with inherited disease) (24) and gnomAD, but is aligned to GRCh37, not automatically integrated into COSMIC, uses its own definition of “mutation significance” rather than drawing on existing equivalent efforts [such as OncoKB (25)] and confusingly combines definitions from ClinVar that were intended for germline variants. We would certainly suggest that COSMIC consider updating, refining, integrating this effort and consider using it as a filtration step for curated variants.

The overrepresentation of variants, and particularly unique population-specific variants, corresponding to the African / African-American ancestral population is also strongly concerning for inadvertent bias. This finding is likely associated with the known phenomenon of decreased linkage disequilibrium and increased occurrence of variants in African ancestral populations (26–28). We observed no significant difference in proportion of misclassified variants among proportion of variants in GnomAD across populations, suggesting that the number of misclassified variants in a population relates to the total number of variants present. Projects such as the Human Pangenome Reference demonstrate the limitations of the current reference genome and opportunities in moving to genomic references with greater diversity, with “3.7 million additional single-nucleotide polymorphisms (SNPs) in regions non-syntenic to GRCh38” among other expansions (29). From a clinical standpoint, moving towards these comprehensive reference efforts at a rate much faster than the transition from GRCh37 to GRCh38 may have the opportunity to adequately serve more patients.

There are limitations to this work we wish to acknowledge. Our overlap search across COSMIC and GnomAD is currently limited to single nucleotide variants. As broad population-level data for structural variants, mutational signatures, and chromosome-scale changes becomes more widely available in future, this could easily be incorporated into the same framework. From the variant to the gene level, it would be fair to draw comparisons across other cancer driver gene datasets. It would also be ideal to expand and streamline this analysis in future across other germline and somatic variant classification and annotation databases (such as ClinVar and OncoKB) (25). One effort examined overlapping variants between GnomAD and The Cancer Genome Atlas, but intentionally focused on rare population variants to study potential biological etiologies including statistical chance, convergent evolution, and correlated mutational rates at specific genetic sites (30). Realistically, the need to apply this search across multiple germline and somatic databases reflects an ongoing limitation of the field regarding data siloing, and the issue of adequate population representation remains active across all of these (31).

The variants that we identified in this study that are misclassified as somatic when actually germline underscores the need for ongoing efforts to improve inclusivity of genetic data across diverse ancestral populations. As we demonstrate, by correctly identifying variants linked to disease as opposed to population, this effort directly offers benefit to all oncology patients.

## Data availability statement

Publicly available datasets were analyzed in this study. Direct links to data are provided in manuscript.

## Author contributions

RP: Conceptualization, Data curation, Formal analysis, Visualization, Writing – original draft, Writing – review & editing. MW: Conceptualization, Data curation, Formal analysis, Supervision, Writing – original draft. PR: Formal analysis, Supervision, Visualization, Writing – review & editing.
